# Dataset on continuous passages of *Trypanosoma brucei* in different laboratory animals

**DOI:** 10.1016/j.dib.2017.08.022

**Published:** 2017-08-31

**Authors:** Paul O. Odeniran, Isaiah O. Ademola

**Affiliations:** Department of Veterinary Parasitology, University of Ibadan, Nigeria

**Keywords:** *Trypanosoma brucei*, Survival time, Laboratory animals, Virulence

## Abstract

Scientist in developing countries maintain trypanosomes in laboratory animals for *in vivo* experiments. We generated data on the adaptation of *Trypanosoma brucei* (NITR201 strain) in balb/c mice (forty-five, 18–23 g), wistar rats (fifteen, 180–220 g) and New Zealand white and chinchilla rabbits (fifteen, 2.8–3.0 kg) in a controlled experimental system. The weight, haematological parameters, course of parasitaemia, temperature, mean survival time and survival proportions of laboratory animals in groups A–E were collected and analysed for differences in response to the same challenge of quantity, strain and species of *T. brucei*. Trypanosome pleomorphism of long, intermediate to short-stumpy forms were among the dataset counts for parasitaemia. Statistical data after analysis were summarised in the supplementary file to show the differences and corresponding reaction of multiple passages.

**Specifications Table**

**Table:** 

Subject area	*Biology, Parasitology*
More specific subject area	*Trypanosomes in laboratory animals*
Type of data	*2 Tables and 4 figures*
How data was acquired	*Microscope (Olympus), Assessment of trypanosomes using rapid matching method and Observation (counting days)*
Data format	*Raw, Analysed*
Experimental factors	*Group A (fifteen rabbits), Group B (fifteen rats), Group C (fifteen mice), Group D (fifteen mice) and Group E (fifteen mice). All experiment done at room temperature. Blood thin films were fixed in absolute methanol and stained with 10% Giemsa stain.*
Experimental features	*Following ethical approval, experiment was conducted in line with guidelines. The survival period and haematological parameters of different laboratory animals was assessed at each stage of passaging using STDM (Standard Trypanosome Detection Method) and Manual differential blood cell counter respectively under a light microscope (Olympus*^*®*^*).*
Data source location	*Nigeria (University of Ibadan Veterinary Parasitology Research Laboratory)*
Data accessibility	*Data available within this article*
Related research article	*Not applicable*

**Value of the data**

• The haematological data and survival time varied among different laboratory animals, and it will be useful in carefully selecting the appropriate host to maintain *Trypanosoma species* for any experimental design.
• Understanding the physiological responses of the laboratory animal hosts could suggest different human responses to *Trypanosoma brucei* infection.
• Different percentage weight gain and loss including severe pathologic responses could discourage further use of some animal models in large quantity.
• Differences in the physiology of individual laboratory animal could affect the pathogenicity and virulence of trypanosome organism during passages.
• This data allows other researchers to extend statistical analysis on experiments related to trypanosomes in the laboratory as this is becoming increasingly needful in developing countries.

## 1 Data

**Fig. 1:**
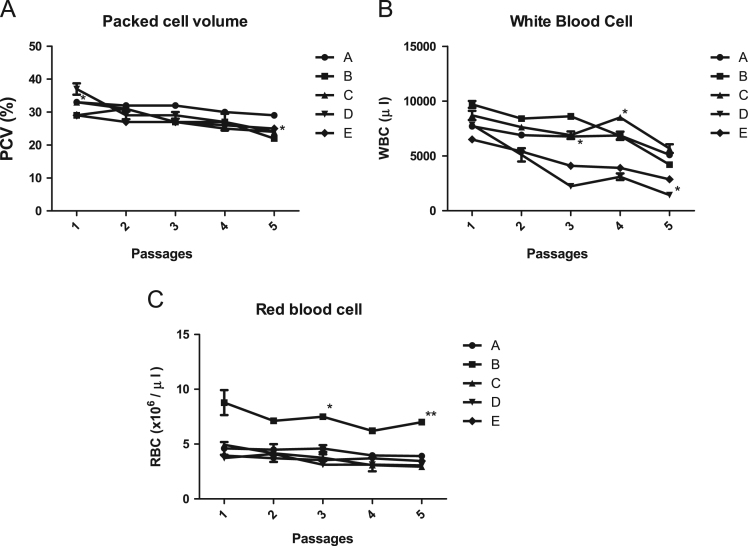
Shows the haematological parameters of laboratory animals passaged with *T. brucei*. A. Detailed packed cell volume data, B. white blood cell data and C. red blood cell data from five passages.

**Fig. 2:**
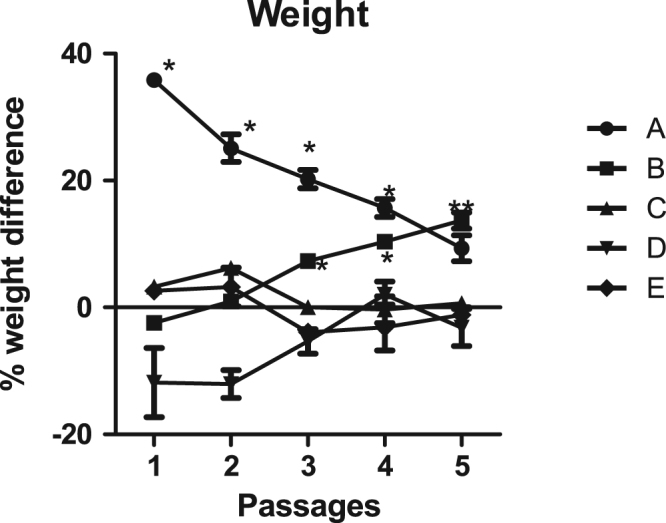
Percentage weight difference of laboratory animals at the peak of parasitaemia for different passages.

**Fig. 3:**
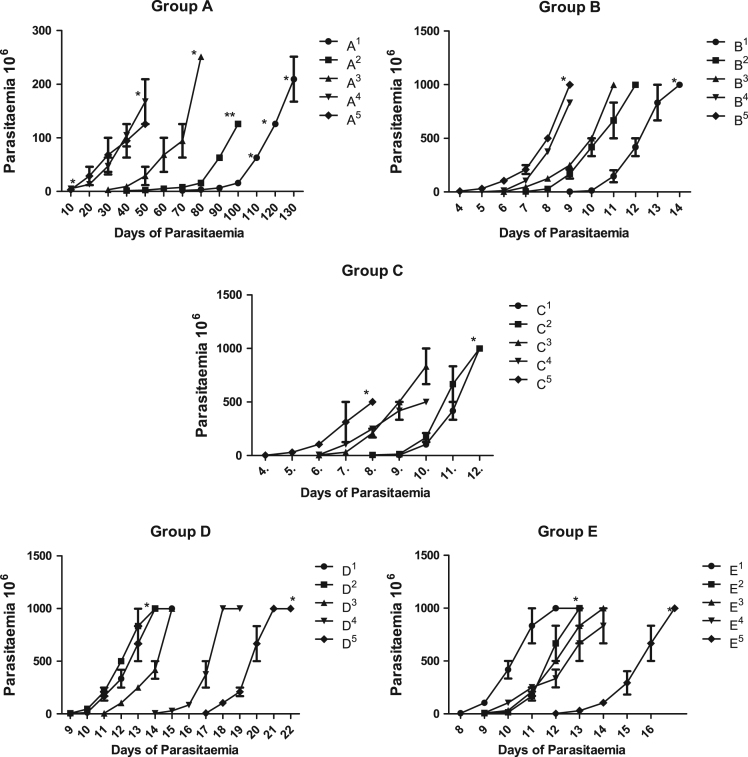
Course of parasitaemia over five passages of the groups. Each point represents the mean±SEM.

**Fig. 4:**
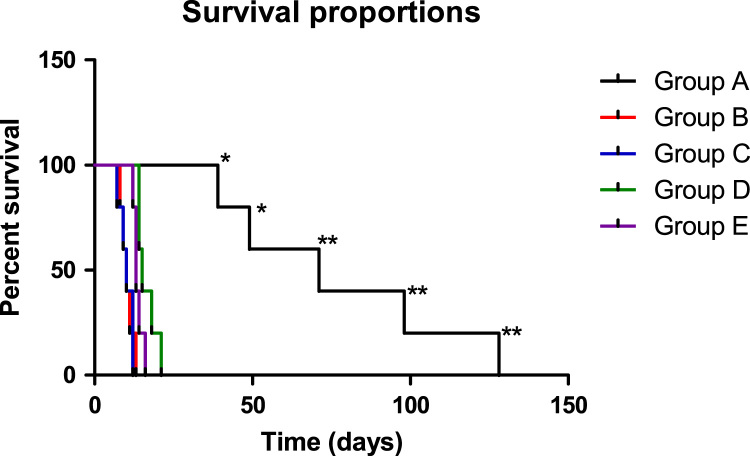
Log-rank Mantel-Cox Test of survival proportions for laboratory animals infected with *Trypanosoma brucei*.

The dataset of this article provides an extensive information on physiologic and haematologic parameters associated with trypanosome continuous passaging in three laboratory animals. Analysed data on differences of the animal physiology to trypanosome is in Fig. 1, Fig. 2, Fig. 3, Fig. 4. Table 1, Table 2 show mean survival and temperature of each group of animals respectively. Analyses were done with Tukey multiple comparison test using GraphPad Prism version 5 for Windows. Survival proportion analysis was done by Log-rank Mantel-Cox Test.

**Table 1:** Mean survival time of infected laboratory animals.

Passages	1		2	3	4	5	Average
Groups	(Day)±(SD)						
A	127±0.43	97.37±0.34		70.31±0.80	48.35±0.52	38.34±0.81	71
B	13.33±0.93	11.33±1.00		10.33±1.04	9±1.00	8.33±1.33	10
C	11.67±0.89	12±0.87		10±0.95	9.33±1.08	7±1.16	10
D	14.33±0.90	14±0.80		14.67±0.80	18.33±0.81	21.33±0.75	15
E	12.33±0.43	12.67±0.85		13.33±0.93	14±0.80	16.33±0.85	13

**Table 2:** Average temperature (°C) at parasitaemia peak.

Group	Passages (Temp °C)				
	**1**	**2**	**3**	**4**	**5**
A	39.6	39.2	39.2	39.1	38.9
B	36.5	36.8	36.6	37.5	37.7
C	36.9	37.1	37.1	37.3	37.3
D	37.2	37.2	37.2	37.3	37.4
E	37.1	37.2	37.2	37.4	37.4

## 2 Experimental design, materials and methods

Datasets were generated with the permission and guidelines of the University of Ibadan animal ethics committee (UI-ACUREC/App/2015/019). Three groups of laboratory animals A, B and C with fifteen rabbits, fifteen rats and fifteen mice respectively, with each group divided into five treatments of three animals each in a treatment per passages (i.e. A^1^–A^5^ representing first to the fifth passages). Each treatment was kept in a transparent netted cage (30 × 45 cm) which allows proper ventilation. All the laboratory animals were fed ad-libitum with pelletized rodent feed and clean water, they were acclimatized for two weeks. Infective blood with *Trypanosoma brucei* was obtained from an infected donor Swiss albino mouse (from the Institute of Advanced Malaria Research, College of Medicine, University of Ibadan, Nigeria). After the proper dilution factor (1:10), 0.2 mL containing 10^5^ of trypanosomes was inoculated intraperitoneally to infect the laboratory animals.

At peak parasitaemia of first passage, trypanosomes were assessed and inoculated into the next sets of animals for the second passage, until the fifth passage was achieved. Groups D and E contains fifteen mice each, these were infected with *T. brucei* from rabbits (Group A) and rats (Group B) respectively. Group A (rabbits) at the peak of parasitaemia were inoculated into group D (i.e. D^1^ – first passage from A^1^ to D^5^ – fifth passage from A^5^), while group E is produced from successive passages from group B (rat) in the same manner. Wet films of the blood from the *T. brucei* infected mice were made under a 7 × 22 mm cover glass. The films were examined under ×400 magnification, and a field was chosen in which the cells were evenly distributed. Giemsa-stained thin smears of blood were also prepared to observe diverse types of pleomorphic trypanosomes and white blood cells present. Parasitaemia was monitored daily from equivalent log number of organisms per millimeter of blood as described by Herbert and Lumsden [1].

Haematological analysis was based on method described [2], and Packed cell volume (PCV) was measured using micro haematocrit method. White blood cell count (WBC) and red blood cells (RBC) were determined in a coulter counter (Cyan Haemocytometer, Belgium), and temperature monitored using digital thermometer. The weight of all the animals have been included in the dataset supplementary files for assessment. Percentage weight difference dataset was necessary to compare the differences between separate groups (Fig. 2).
