# Supplementary material for: Dataset on continuous passages of Trypanosoma brucei in different laboratory animals
Source: Data Brief. 2017 Aug 31;14:629–34. doi: 10.1016/j.dib.2017.08.022 (PMC5587882; doi:10.1016/j.dib.2017.08.022)
Supplement: Supplementary file 1 — Transparency document [file mmc2.docx]

Author’s agreement

The authors declare that we have no conflict of interest and we have agreed to publish this manuscript with Data in brief.


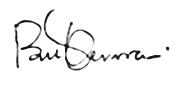


Dr. I.O. Ademola Dr. P. O. Odeniran
